# β1 integrin mediates unresponsiveness to PI3Kα inhibition for radiochemosensitization of 3D HNSCC models

**DOI:** 10.1016/j.biopha.2024.116217

**Published:** 2024-01-28

**Authors:** Irina Korovina, Marc Elser, Olegs Borodins, Michael Seifert, Henning Willers, Nils Cordes

**Affiliations:** a OncoRay – National Center for Radiation Research in Oncology, Faculty of Medicine Carl Gustav Carus, Technische Universität Dresden, Dresden, Germany; b Helmholtz-Zentrum Dresden - Rossendorf, Institute of Radiooncology – OncoRay, Dresden, Germany; c Institute for Medical Informatics and Biometry (IMB), Faculty of Medicine Carl Gustav Carus, Technische Universität Dresden, Dresden, Germany; d National Center for Tumor Diseases (NCT), Partner Site Dresden, German Cancer Research Center (DKFZ), Heidelberg, Germany; e Department of Radiation Oncology, Massachusetts General Hospital, Harvard Medical School, Boston, MA, USA; f German Cancer Consortium (DKTK), Partner Site Dresden, and German Cancer Research Center (DKFZ), Heidelberg, Germany; g Department of Radiotherapy and Radiation Oncology, University Hospital Carl Gustav Carus, Technische Universität Dresden, Dresden, Germany

**Keywords:** Head and neck cancer, Radio(chemo)sensitization, PI3K inhibitors, MAPK signaling, β1 integrin

## Abstract

Phosphoinositide 3-kinase (PI3K)-α represents a key intracellular signal transducer involved in the regulation of key cell functions such as cell survival and proliferation. Excessive activation of PI3Kα is considered one of the major determinants of cancer therapy resistance. Despite preclinical and clinical evaluation of PI3Kα inhibitors in various tumor entities, including head and neck squamous cell carcinoma (HNSCC), it remains elusive how conventional radiochemotherapy can be enhanced by concurrent PI3K inhibitors and how PI3K deactivation mechanistically exerts its effects. Here, we investigated the radiochemosensitizing potential and adaptation mechanisms of four PI3K inhibitors, Alpelisib, Copanlisib, AZD8186, and Idelalisib in eight HNSCC models grown under physiological, three-dimensional matrix conditions. We demonstrate that Alpelisib, Copanlisib and AZD8186 but not Idelalisib enhance radio- and radiochemosensitivity in the majority of HNSCC cell models (= responders) in a manner independent of *PIK3CA* mutation status. However, Alpelisib promotes MAPK signaling in non-responders compared to responders without profound impact on Akt, NFκB, TGFβ, JAK/STAT signaling and DNA repair. Bioinformatic analyses identified unique gene mutations associated with extracellular matrix to be more frequent in non-responder cell models than in responders. Finally, we demonstrate that targeting of the cell adhesion molecule β1 integrin on top of Alpelisib sensitizes non-responders to radiochemotherapy. Taken together, our study demonstrates the sensitizing potential of Alpelisib and other PI3K inhibitors in HNSCC models and uncovers a novel β1 integrin-dependent mechanism that may prove useful in overcoming resistance to PI3K inhibitors.

## 1. Introduction

Head and neck squamous cell carcinoma (HNSCC) represents the sixth most common cancer worldwide [1]. To date, the principal treatment strategies for HNSCC include combinations of surgical resection, radiotherapy, chemotherapy, and targeted therapy (Cetuximab). Despite these broad options, 5-year survival rates for patients with locally advanced HNSCC have improved only moderately during the last thirty years and remain around 50% [1,2].

Unraveling the mutational landscape of a large variety of cancer types has paved the way for molecular targeted approaches including those targeting the various forms of phosphoinositide 3-kinases (PI3K). PI3K signaling plays a pivotal role in the regulation of various cellular properties including survival, proliferation, migration and cell cycle progression and is tightly controlled in normal cells [3–5]. In cancers, and in particular in HNSCC, the PI3K pathway is the most frequently mutated oncogenic pathway with a frequency of approximately 30% [6]. Moreover, hyperactivation of PI3K signaling has been observed in approximately 90% of HNSCC cases, which is associated with cancer progression and therapy resistance [7]. Thus, PI3K targeting represents a promising therapeutic tool to improve conventional cancer therapy. Several pan-PI3K (e.g. Copanlisib, BKM-120, PX-866) and isoform-selective PI3K (e.g. Alpelisib, CYH33, AZD8186, Idelalisib) inhibitors elicit significant anti-tumor activity in HNSCC and other tumor entities [3,4]. However, despite these encouraging results, intrinsic and acquired resistance of cancer cells to PI3K inhibition substantially limit therapy efficacy, which might be related to genetic, epigenetic and microenvironmental factors [8].

Based on our experience with other small molecule inhibitors, where we have observed a heterogeneous response to the drug, we hypothesized that HNSCC models would also exhibit heterogeneity with respect to the effect of PI3K inhibitors. We further postulated that a group of non-responding HNSCC models represents patients who either (a) would not benefit from a PI3K therapy, or (b) would benefit from a multi-targeting approach that simultaneously impairs PI3K bypass signaling. In 3D extracellular matrix (ECM)-embedded HNSCC cultures, we show that the PI3K inhibitors Alpelisib, Copanlisib and AZD8186 induce sensitization to radio- and radiochemotherapy in the majority of cell models (= responders). We further demonstrate that, based on bioinformatic analyses, a particular set of ECM associated genes is mutated in non-responders indicating a therapeutically exploitable vulnerability. By targeting the adhesion receptor β1 integrin, we are able to overcome PI3K inhibitor resistance. Overall, our data nominate PI3Kα as a promising target to overcome radio(chemo)resistance in HNSCC and propose a potential treatment strategy to improve the efficacy of conventional therapy by simultaneous blockage of β1 integrin/PI3Kα signaling.

## 2. Materials and methods

### 2.1. Cell culture

HNSCC cell lines (Cal33, FaDu, HSC4, SAS, UTSCC5, UTSCC8, UTSCC14, UTSCC15) were kindly provided by R. Grenman (Turku University Central Hospital, Turku, Finland). All cells were grown in Dulbecco’s Modified Eagle’s Medium (DMEM) high glucose (Sigma-Aldrich) supplemented with 10% heat-inactivated fetal bovine serum (Sigma-Aldrich), and 1x MEM non-essential amino acids (Thermo Fischer Scientific) at 37 °C in a humidified 8.5% CO2 incubator. For 3D cell cultures, cells were embedded in 0.5 mg/mL laminin-rich extracellular matrix (lrECM) (Corning) as previously described [9]. Experiments were performed using asynchronously growing cells within passages 2 – 4. All cell lines were STR-authenticated and regularly monitored for mycoplasma contamination using Venor^®^GeM OneStep Mycoplasma Detection Kit (Minerva Biolabs).

### 2.2. Chemicals and treatments

Alpelisib (MedChemExpress), AZD8186 (MedChemExpress), Idelalisib (MedChemExpress), SP600125 (MedChemExpress), SCH772984 (MedChemExpress), Cilengitide (MedChemExpress), E7820 (Biozol), BTT3033 (Tocris Bioscience), and Ralimetinib (LY2228820) dimesylate (Cayman Chemical) were dissolved in dimethyl sulfoxide (DMSO, AppliChem). Copanlisib (MedChemExpress) was dissolved in 1 M HCl. Cisplatin was obtained from the pharmacy of University Hospital Carl Gustav Carus, Dresden. The inhibitory β1 integrin antibody AIIB2 was isolated and purified from a human choriocarcinoma hybridoma [10]. Nonspecific rat IgG1 was purchased from Santa Cruz Biotechnology. Inhibitors were applied at concentrations, which result in reduction of clonogenic survival ≤ 25%: Cisplatin (FaDu – 0.1 µM; Cal33, SAS, HSC4, UTSCC5, UTSCC8, UTSCC14 and UTSCC15 – 0.5 µM); Alpelisib (Cal33 – 0.2 µM; UTSCC8 – 0.5 µM; UTSCC5 – 1 µM; FaDu, HSC4, SAS, UTSCC14, UTSCC15 – 2 µM); Copanlisib (Cal33 – 2 nM; FaDu, HSC4, UTSCC5, UTSCC8, UTSCC14, UTSCC15 – 30 nM); AZD8186 – 1.5 µM; Idelalisib – 0.5 µM, 1 µM and 2 µM; SP600125 – 5 µM; Ralimetinib – 0.5 µM; SCH772984 – 0.1 µM; E7820 – 0.3 µM; BTT3033 – 20 µM; Cilengitide – 20 µM. The inhibitory β1 integrin antibody AIIB2 was applied at a concentration of 10 µg/mL.

### 2.3. 3D colony formation assay

The 3D colony formation assay was utilized to assess the ability of a single cell to form a colony as previously published [9]. Briefly, HNSCC cells were embedded into 0.5 mg/mL lrECM in 96-well plates pre-covered with 1% agarose. After 24 h, cells were treated with drugs and 1 h later irradiated with 6 Gy X-rays using Yxlon Y.TU 320 (Yxlon Int. GmbH), and 24 h later the growth medium was replaced with the fresh one. In the experiments with integrin inhibitors, treatment strategy was conducted as follows: E7820, BTT3033, Cilengitide and AIIB2 were added to 3D matrix-embedded HNSCC cell cultures, 1 h later Cisplatin and/or Alpelisib were applied and after another 1 h cells were irradiated with 6 Gy X-rays. After a cell line-dependent growth period, cell colonies were fixed with 9% formaldehyde solution in PBS. Colonies > 50 µm in size were counted using Axiovert 25 Inverted Light Microscope (Carl Zeiss GmbH). Plating efficiencies (the number of counted colonies divided by the number of seeded cells) were calculated for all treatment groups and divided by the plating efficiency of non-irradiated vehicle control (set as 100%).

### 2.4. DNA isolation

HNSCC cells were grown in 0.5 mg/mL lrECM for 4 days [11]. Next, cells were segregated using Trypsin/EDTA solution (Sigma-Aldrich) and DNA was isolated using the NucleoSpin^®^ Tissue kit (Macherey-Nagel) according to the manufacturer’s protocol. DNA concentration was measured with NanoDrop One^C^ Spectrophotometer (Thermo Fisher Scientific) and 1 μg of DNA was utilized for the whole exome sequencing.

### 2.5. Whole exome sequencing

Whole exome sequencing (WES) procedure for FaDu, SAS, UTSCC14 and UTSCC15 cell lines was described in our previous publication [11]. Analysis of Cal33, HSC4, UTSCC5 and UTSCC8 cell lines was performed as follows. Genomic DNA (1 μg) was sheared to 100 – 400 bp using a Covaris S2 system (Covaris). Sheared DNA was subjected to Illumina paired-end DNA library preparation. Next, the libraries were enriched for target sequences using the SureSelect XT Human All Exon v7 (Agilent Technologies) according to the manufacturer’s recommendations. Enriched libraries were sequenced using the NovaSeq 6000 platform (Illumina) on an S1 200 cycles flowcell, generating 35 Mio 100 bp paired-end fragments on average (100x coverage).

### 2.6. Bioinformatics pipeline

Quality control of raw WES data of HNSCC cell lines was done by FastQC v0.11.4 [11]. Adapter removal and trimming of reads was performed using TrimGalore v0.4.2. Mapping of reads against the human genome reference sequence (GRCh37 release 13) was done by BWA-MEM v0.7.13 with standard settings and duplicates were marked using Samblaster v0.1.24. Mapped reads were locally aligned with the Genome Analysis Toolkit (GATK 3.5, tools: RealignerTargetCreator, IndelRealigner, BaseRecalibrator, PrintReads). Alignment summary metrics were determined with Picard tools v1.141 and SAMtools v1.3. Mutect2 was used for variant calling. Additional filtering and annotation of the predicted variants was done with Annovar v1Feb2016. The predicted variants were annotated using ClinVar, COSMIC v.94, avsnp150, ExAC_nontcga and gnomAD_exome variant information. For each cell line, only exonic, protein-altering mutations that were independently predicted in all three technical replicates of the WES data of a cell line were used for subsequent analysis.

### 2.7. Western blotting

Protein isolation from 3D HNSCC cell cultures was performed using ice-cold RIPA lysis buffer (50 mM Tris-HCL pH 7.4, 1% NP-40, 0.25% Na-deoxycholate, 150 mM NaCl, 1 mM EDTA) supplemented with COMPLETE^™^ protease inhibitor cocktail (Hoffmann-La Roche), 1 mM Na-Orthovanadate and 2 mM Na-Fluorid as published [12]. At equal amounts, proteins were subjected to SDS-PAGE/Western blotting. Next, nitrocellulose membranes (0.2 µM, Cytiva) were incubated with indicated primary antibodies, followed by incubation with horseradish-peroxidase conjugated secondary antibodies. Primary antibodies used in Western blot analysis are phospho-Akt (Ser473) (1:1000, Cell Signaling Technology Cat# 4060, RRID:AB_2315049), Akt (1:1000, Cell Signaling Technology Cat# 9272, RRID:AB_329827), phospho-4E-BP1 (Ser65) (1:1000, Cell Signaling Technology Cat# 9456, RRID:AB_823413), 4E-BP1 (1:1000, Cell Signaling Technology Cat# 9644, RRID:AB_2097841), phospho-GSK-3β (Ser9) (1:1000, Cell Signaling Technology Cat# 9336, RRID:AB_331405), GSK-3β (1:1000, Cell Signaling Technology Cat# 9832, RRID:AB_10839406), phospho-DNA-PK (Ser2056) (1:600, Abcam Cat# ab18192, RRID: AB_869495), DNA-PK (1:1000, Cell Signaling Technology Cat# 4602, RRID:AB_10692482), phospho-ATM (Ser1981) (1:1000, Cell Signaling Technology Cat# 4526, RRID:AB_2062663), ATM (1:1000, Cell Signaling Technology Cat# 2873, RRID:AB_2062659), phospho-ATR (Thr1989) (1:1000, Cell Signaling Technology Cat# 30632, RRID: AB_2798992), phospho-ATR (Ser428) (1:1000, Thermo Fisher Scientific Cat# PA5–121295, RRID:AB_2914867), ATR (1:1000, Thermo Fisher Scientific Cat# PA5–85507, RRID:AB_2792647), vinculin (1:4000, Sigma-Aldrich Cat# V9131, RRID:AB_477629), GAPDH (1:2000, Cell Signaling Technology Cat# 2118, RRID:AB_561053). Secondary antibodies used include rabbit IgG horseradish-peroxidase linked whole antibody (1:5000, Cytiva Cat# NA934, RRID:AB_772206) and mouse IgG horseradish-peroxidase linked whole antibody (1:5000, Cytiva Cat# NXA931, RRID:AB_772209). Protein detection was performed using enhanced-chemiluminescence (ECL) detection reagent (GE Healthcare) and Fusion FX (Vilber Lourmat GmbH) followed by Fiji software-based densitometry. Phosphorylated forms and the corresponding total proteins for each experiment were detected in separate blots. In order to normalize expressions of proteins of interest, all blots were reprobed with loading control antibodies (GAPDH or vinculin). All Western blot data are presented as a ratio of normalized phospho-protein levels to normalized total protein levels.

### 2.8. Akt activity assay

UTSCC14, Cal33 and FaDu cell lines were embedded in 0.5 mg/mL lrECM and treated with Alpelisib and/or Cisplatin and irradiated with 6 Gy X-rays. After 1 h crude cell lysates were prepared and Akt activity in 10 µg of protein lysates was analyzed using Akt Kinase Activity Kit (Enzo Life Science) according to the manufactureŕs instructions.

### 2.9. 3D foci assay

3D foci assay was performed as published [13]. In brief, HNSCC cells were embedded in 0.5 mg/mL lrECM and treated with Alpelisib and/or X-rays (3 Gy X-rays – for γH2A.X and Rad51 foci; 6 Gy X-rays – for 53BP1 foci). After 24 h, cells were segregated using Trypsin/EDTA solution (Sigma-Aldrich) and fixed with 3% formaldehyde/PBS. Next, cells were permeabilized with 0.25% Triton-X-100/PBS and blocked with 1% BSA/PBS. Incubation with primary antibodies was performed overnight at 4 °C following 2.5 h incubation with secondary antibodies conjugated with Alexa Fluor 488 (1:500, Thermo Fisher Scientific Cat# A-11034, RRID:AB_2576217), Alexa Fluor 546 (1:500, Thermo Fisher Scientific Cat# A-11010, RRID:AB_2534077) and Alexa Fluor 633 (1:500, Thermo Fisher Scientific Cat# A-21126, RRID:AB_2535768). Primary antibodies used are 53BP1 (1:200, Novus Cat# NB100–304, RRID:AB_10003037), Rad51 (1:200, Millipore Cat# PC130, RRID:AB_2238184) and phospho-Histone H2A.X (Ser139) (1:200, Millipore Cat# 05–636, RRID: AB_309864). Samples were embedded in fluorescence mounting medium (Dako). Images of 53BP1 foci were acquired using an Axio Imager M1 microscope (Carl Zeiss GmbH), images of Rad51 and γH2A.X foci were obtained using LSM980 with Airyscan 2 Confocal Microscope (Carl Zeiss GmbH). Foci analysis was performed using Fiji software.

### 2.10. Phosphorylation pathway analysis

Phosphorylation measurements of Akt, MAPK, JAK/STAT, NFκB and TGFβ pathways from 3D HNSCC cell cultures irradiated with 6 Gy X-rays and treated with Alpelisib (DMSO as a control) were performed using Human Phosphorylation Pathway Profiling Array C55 (RayBiotech) according to the manufactureŕs instructions. Phospho-array analysis was performed using Fiji software.

### 2.11. RNA expression data

We employed Gene Expression Omnibus (GEO) to obtained the raw RNA sequencing data for three HNSCC cell models: Cal33, FaDu, and UTSCC14 (accession numbers: GSM4272668, GSM4272669, GSM1716435, GSM4617286). To ensure robust and consistent analysis, all data sets underwent standard processing using R, leveraging the following packages: GEOquery, tidyverse, dplyr, TCGAbiolinks, sesame, and maftools. Next, we normalized the data by converting them into TPM+ 1 (Transcripts Per Million plus one) to facilitate unbiased comparison. To identify genes with similar expression levels between Cal33 and FaDu cell models, we performed a comparative analysis of their respective gene expression profiles. This approach allowed us to identify genes that displayed comparable transcript abundance in these two cell lines. Subsequently, we integrated these data with the gene expression data from the UTSCC14 cell model, enabling a more comprehensive assessment of gene expression patterns. Through this integration, we identified differentially expressed genes (DEG) across the cell models, thereby revealing potential molecular distinctions among them.

### 2.12. Gene enrichment and network analyses

All enrichment and protein-protein interaction network analyses were conducted through the CytoScape platform (v3.4.0) [14]. The initial network was constructed by combining WES and RNA expression datasets for responder (UTSCC14) and non-responder (Cal33, FaDu) cell models using STRING (75% confidence) [15]. Afterwards, the network was enriched with *PIK3CA* and *ITGB1* and their physical interactors acquired from the Biological General Repository for Interaction Datasets (BioGRID) database (https://thebiogrid.org). Additionally, we incorporated key kinases identified in the Human Phosphorylation Pathway Profiling Array. Subsequently, we selected genes with first neighbor interaction between *ITGB1* and *PIK3CA*. In order to identify genes that respond to Alpelisib/X-ray treatment, we filtered the network based on the following criterion: kinases with at least 40% differences in phosphorylation between non-responder (Cal33) and responder (UTSCC14) cells after Alpelisib/X-rays. Finally, using GeneMANIA [16], we reconstructed protein-protein network to identify a key interactor within the formed network on the basis of physical interactions, co-expression, predicted colocalization, shared pathways, genetic interaction, and shared protein domains.

### 2.13. Functional enrichment analysis

Functional pathway enrichment analysis of the final network was performed via ShinyGO and ClueGO for Gene Ontology (GO) and Kyoto Encyclopedia of Genes and Genomes (KEGG) [17–20]. Exome functional pathway enrichment analysis was performed via SNPnexus tool for Reactome [21].

### 2.14. Statistical analysis

Data represent mean ± SEM values calculated on at least three independent experiments. Normalization methods are described in each respective Material and Methods section. Statistical significance between two groups was calculated using two-tailed unpaired Student t test. For multiple comparisons, one-way ANOVA (followed by Dunnett or Sidak post hoc test) was applied. Differences were considered statistically significant at * , *P* < 0.05; * *, *P* < 0.01; * ** , *P* < 0.001; * ** *, *P* < 0.0001.

## 3. Results

PI3Kα inhibition is associated with a heterogeneous response pattern in radiochemotherapy-treated HNSCC cells.

**Fig. 1.**
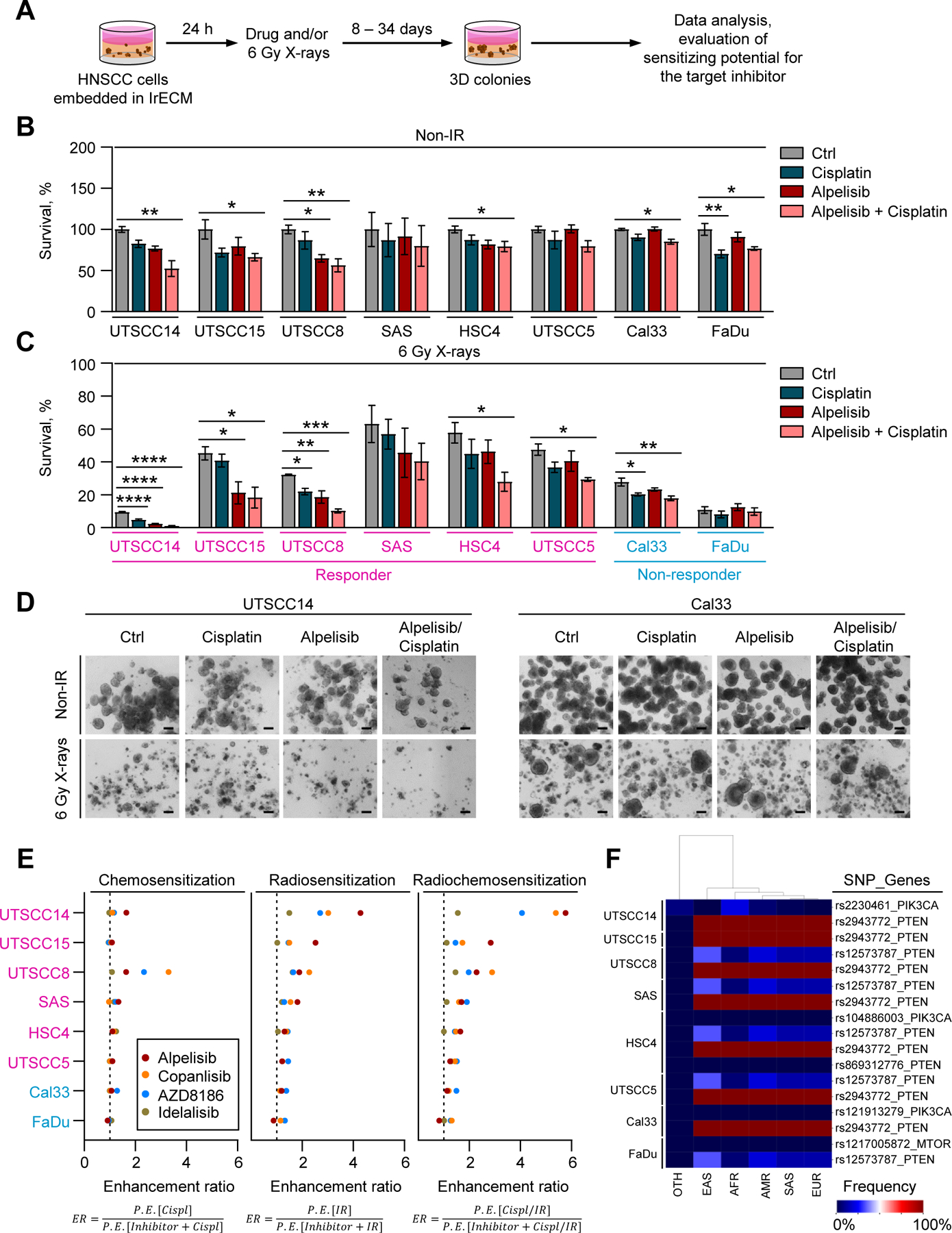
PI3Kα inhibitor Alpelisib sensitizes 3D lrECM HNSCC cell cultures to radiochemotherapy. (A) Human HNSCC cell lines were embedded in 3D laminin-rich extracellular matrix (lrECM) and exposed to PI3K inhibitors (Alpelisib, Copanlisib, AZD8186 or Idelalisib; DMSO or 1 M HCl as a control), Cisplatin and 6 Gy X-ray irradiation. Basal clonogenic survival (B) and clonogenic radiation survival (C) in a panel of HNSCC cell lines treated as indicated. Survival of untreated, non-irradiated cells was set as 100%. Data represent mean ± SEM of at least three independent experiments. Differences were compared using a one-way ANOVA with Dunnett post hoc test; * , *P* < 0.05; * *, *P* < 0.01; * ** , *P* < 0.001. (D) Representative images illustrating 3D colony formation assay of UTSCC14 and Cal33 cells treated with Alpelisib and/or Cisplatin and irradiated with 6 Gy X-rays, scale bar 100 µm. (E) Comparative analysis of the chemo- and radiosensitizing potential of the PI3K inhibitors Alpelisib, Copanlisib, AZD8186, and Idelalisib (2 µM) in the 3D lrECM HNSCC cell culture panel. Data are expressed as enhancement ratios (ER) calculated as indicated. P.E. – plating efficiency. (F) Heatmap illustrating prevalence of *PIK3CA*, *MTOR* and *PTEN* mutations identified by WES in a panel of HNSCC cell lines across different human populations. OTH – other; EAS – East Asian; AFR – African; AMR – Central/South American; SAS – South Asian, EUR – European.

We first investigated the role of PI3K kinases in HNSCC therapy resistance using a 3D clonogenic survival assay. To this end, a panel of eight 3D lrECM-embedded HNSCC models was treated with Alpelisib (selective PI3Kα inhibitor), Copanlisib (pan-PI3K inhibitor), AZD8186 (selective PI3Kβ/δ inhibitor), or Idelalisib (selective PI3Kδ inhibitor) in combination with Cisplatin and/or 6 Gy X-rays (Fig. 1A and S1A, B). Our data revealed moderate changes in basal survival of HNSCC cells for all tested inhibitors relative to controls (Fig. 1B and S1C, D). For X-ray radiation, survival of the majority of cell models was significantly reduced upon PI3K inhibition and further lowered in combination with Cisplatin in a cell line-dependent manner compared to controls (Fig. 1C, D and S1C, D). Comparative analysis of enhancement ratios (ER) was performed to assess the chemo-, radio- and radiochemosensitizing potentials of the tested PI3K inhibitors. We identified Alpelisib and Copanlisib as the most potent agents (Fig. 1E). We continued our experiments with the selective PI3Kα inhibitor Alpelisib, which is expected to have lower clinical toxicity compared with the PI3K pan-inhibitor Copanlisib [4].

**Fig. 2.**
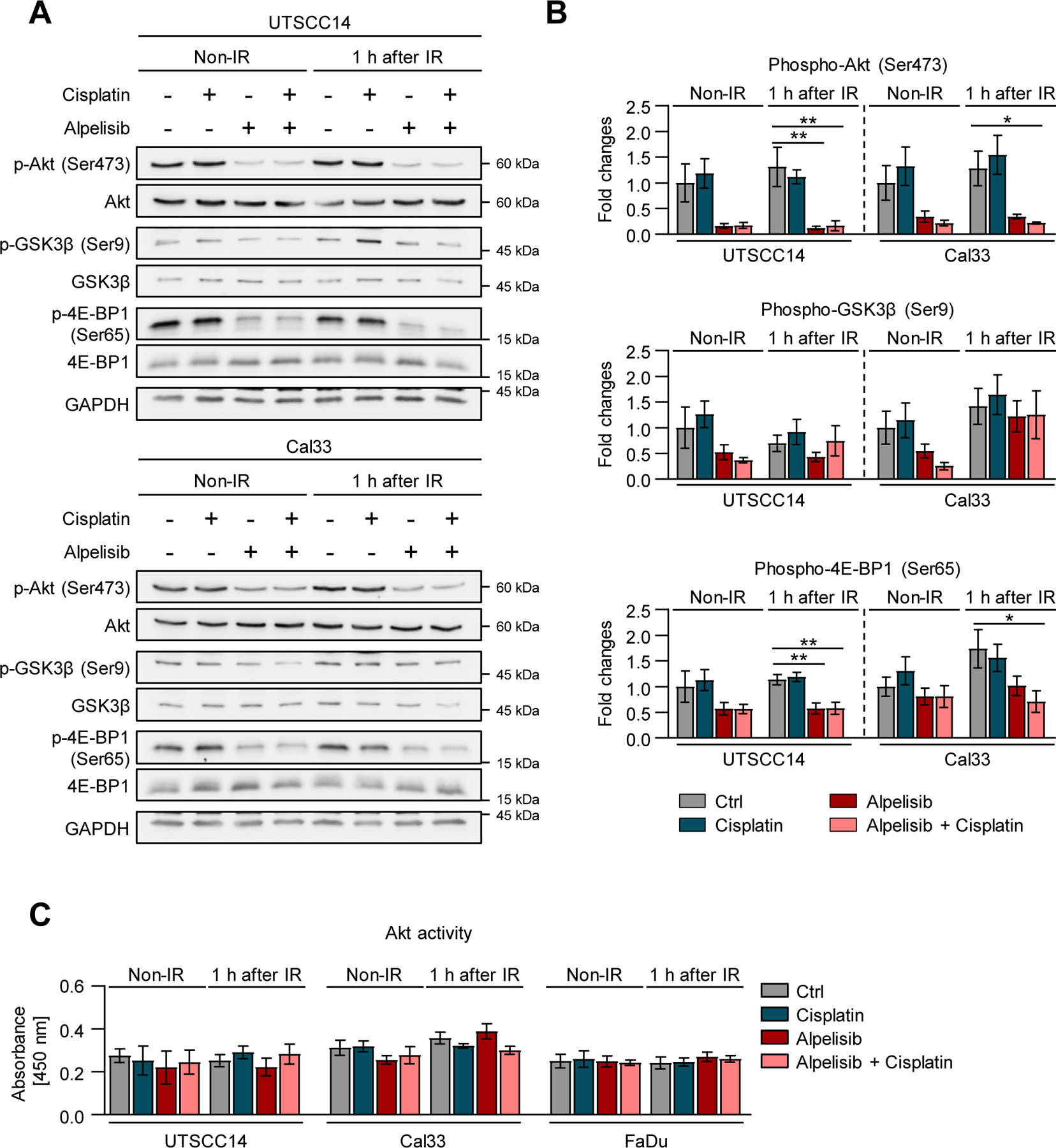
PI3Kα inhibitor Alpelisib downregulates Akt signaling pathway with similar potency in both responder and non-responder HNSCC cell models. (A) Western blot analyses from whole cell lysates of UTSCC14 (responder) and Cal33 (non-responder) cell lines treated with Cisplatin, Alpelisib (DMSO as control) and 6 Gy X-rays (IR) and densitometric quantifications (B) of phosphorylation and total expression levels of indicated proteins. GAPDH was used as a loading control. Data are shown as mean ± SEM (*n* = 4). (C) Akt kinase activity in UTSCC14, Cal33 and FaDu cell lines exposed to indicated PI3K inhibitors (DMSO as a control) with data presented as mean ± SEM (*n* = 3). Statistical analysis in B and C was performed using a one-way ANOVA with Dunnett post hoc test; * , *P* < 0.05; * *, *P* < 0.01.

Next, we aimed to understand the molecular basis of the observed variable radio- and radiochemosensitizing effects of Alpelisib. Whole exome sequencing (WES) failed to show any correlation between sensitizing effects of Alpelisib and mutations in genes encoding key members of the PI3K/Akt pathway (e.g. *PIK3CA*, *MTOR, PTEN*) (Fig. 1F). In terms of PI3K/Akt pathway activity, Alpelisib treatment reduced phosphorylation of Akt (Ser473), GSK3β (Ser9) and 4E-BP1 (Ser65) to a similar extent in responder and non-responder cell models (Fig. 2A, B and S2). Likewise, Akt activity was not significantly modified upon Alpelisib exposure (Fig. 2C). Taken together, our data show that inhibition of PI3Kα kinase causes varying degrees of radiochemosensitization in different HNSCC models and without an obvious mutational biomarker to predict drug effect.

### 3.1. PI3Kα inhibitor Alpelisib does not affect DNA damage repair

**Fig. 3.**
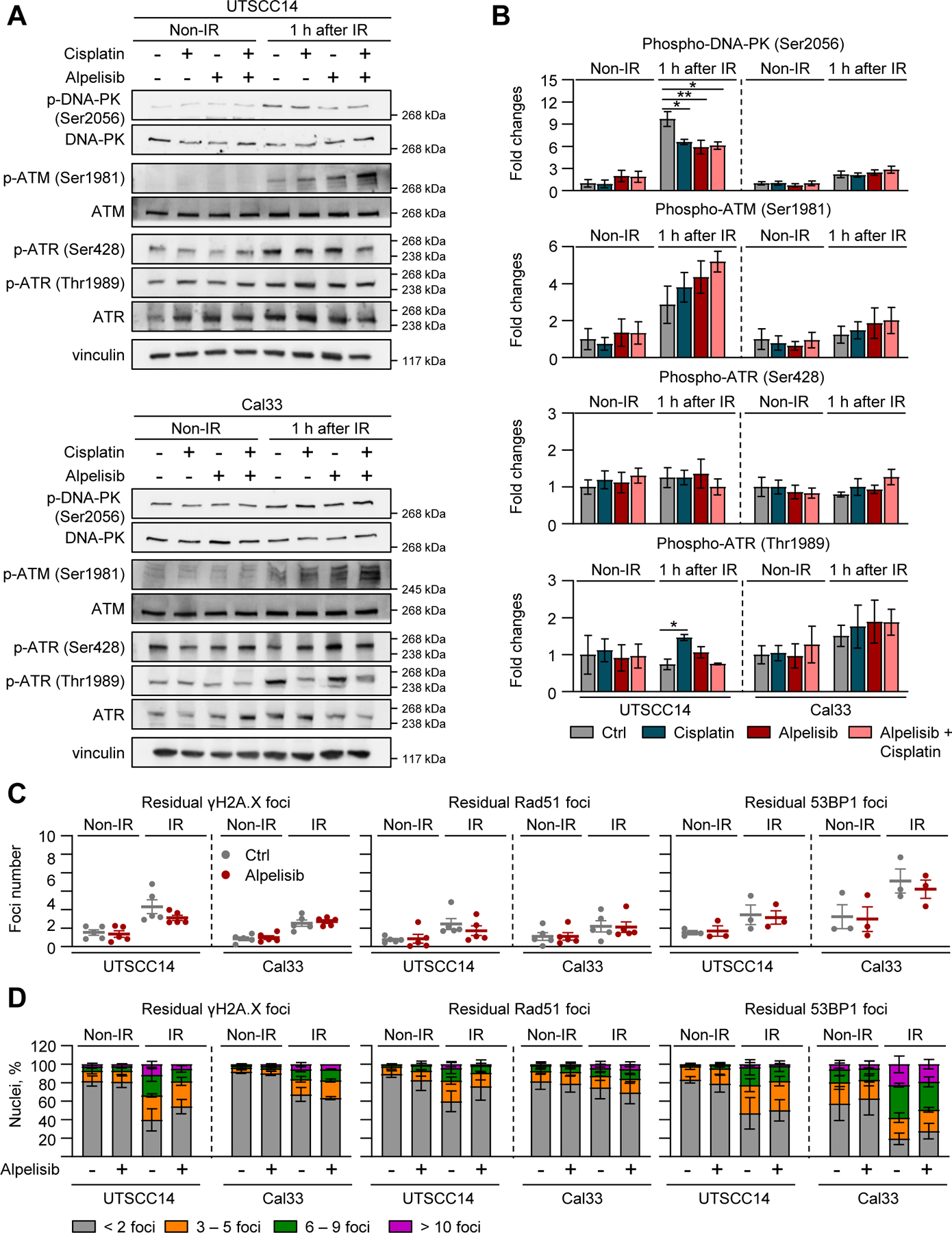
PI3Kα inhibitor Alpelisib does not interfere with DNA damage repair in HNSCC cell lines. (A) Western blot analyses from whole cell lysates of UTSCC14 (responder) and Cal33 (non-responder) cell lines treated with Cisplatin, Alpelisib (DMSO as control) and 6 Gy X-rays (IR) images and densitometric quantifications (B) of phosphorylation and total expression levels of indicated proteins. Vinculin was used as a loading control. Data are shown as mean ± SEM of at least three independent experiments. Residual γH2A.X, Rad51 and 53BP1 foci numbers in total (C) and foci numbers per nuclei (D) in UTSCC14 and Cal33 cell lines treated with Alpelisib and/or X-rays. Data represent mean ± SEM of at least three independent experiments. Statistical analysis in B was performed using a one-way ANOVA with Dunnett post hoc test. Differences in C were analyzed with a two-tailed unpaired Studentś t-test; * , *P* < 0.05; * *, *P* < 0.01.

We next hypothesized that Alpelisib-mediated radiochemosensitization is due to impairment of DNA damage repair. We first evaluated phosphorylation levels of three key regulators of DNA damage, i.e. ATM, DNA-PK and ATR. While basal phospho-levels of DNA-PK (Ser2056), ATM (Ser1981) and ATR (Ser428, Thr1989) remained constant upon mono- and combined therapies (Alpelisib/Cisplatin), responder (UTSCC14) and non-responder (Cal33) models differed markedly in the magnitude of phosphorylation changes of DNA-PK (Ser2056), ATM (Ser1981) and ATR (Thr1989) upon irradiation (Fig. 3A, B). Interestingly, responder and non-responder models revealed non-significant but cell line-dependent differences in the phosphorylation of examined DNA repair enzymes upon Alpelisib/Cisplatin/X-ray combination therapy relative to Cisplatin/X-rays (Fig. 3A, B).

Next, we assessed homologous recombination (HR)- and non-homologous end joining (NHEJ)-dependent DNA double-strand break (DSB) repair capacity by determining residual γH2A.X, Rad51, and 53BP1 foci. Unexpectedly, we found that Alpelisib failed to significantly influence these DSB repair processes with no clear differences between responder and non-responder models (Fig. 3C, D). Taken together, these data suggest that inhibition of the PI3K/Akt pathway is not responsible for radiosensitization of these HNSCC models by DSB repair, despite previous data supporting a role for this pathway in this regard [22].

Alpelisib induces differential phosphorylation patterns in responding and non-responding HNSCC cell models.

**Fig. 4.**
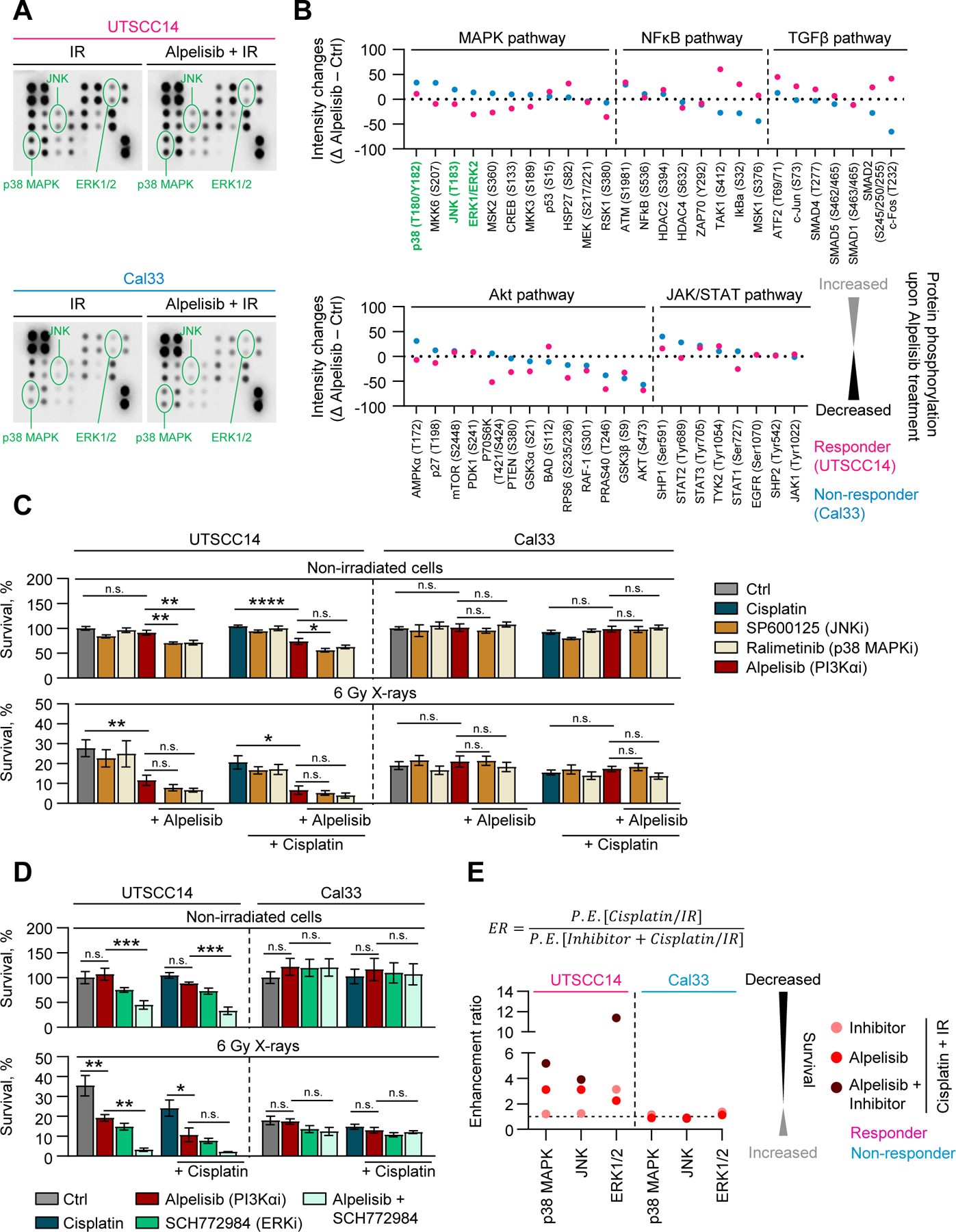
PI3Kα inhibitor Alpelisib elicits differential effects on phosphorylation of different MAPK pathway components in responder and non-responder cell models. (A) Representative images of phosphorylation pathway screen. (B) Phosphorylation profiles of UTSCC14 (responder) and Cal33 (non-responder) cell models 1 h after 6 Gy X-ray radiation (IR) and Alpelisib treatment. Untreated, irradiated cells were used as a control (*n* = 1). (C, D) Basal clonogenic survival and survival upon X-ray radiation in UTSCC14 and Cal33 cell lines treated with Alpelisib in combination with either SP600125, Ralimetinib, SCH772984 or Cisplatin. Survival of untreated, non-irradiated cells was set as 100%. Data represent mean ± SEM (*n* = 4). (E) Enhancement ratios of effects from combined Alpelisib targeting with either SP600125, Ralimetinib or SCH772984 in UTSCC14 and Cal33 cells. Differences in C and D were compared using a one-way ANOVA with Sidak post hoc test; * , *P* < 0.05; * *, *P* < 0.01; * ** , *P* < 0.001; * ** *, *P* < 0.0001; n.s. – non-significant.

To gain insight into the mechanisms underlying Alpelisib’s sensitizing versus non-sensitizing effects, we initially employed phosphorylation arrays to determine phosphorylation changes in key members of Akt, MAPK, JAK/STAT, NFκB, and TGFβ pathways. Our data revealed that Alpelisib generally causes lower phosphorylation levels of MAPK signaling components in responder models (8 out of 11 kinases and transcription factors) compared to non-responder models (Fig. 4A, B). For the latter, Alpelisib induced phosphorylation in 9 out of 11 MAPK associated kinases (Fig. 4A, B). Akt, JAK/STAT, NFκB and TGFβ pathways showed either parallel phosphorylation changes or a greater reduction in phosphorylation in non-responders relative to responders (Fig. 4A, B). Given these findings, we hypothesized that persistent activation of MAPK kinase pathway might be associated with limited efficacy of Alpelisib in non-responder cell models resulting in absence of radiochemosensitization. To address this possibility, we selected pharmacological inhibitors for MAPK signaling components (SP600125, JNK inhibitor; Ralimetinib, p38 MAPK inhibitor; SCH772984, ERK1/2 inhibitor) and examined their potential to mediate cytotoxicity and radiochemosensitization when combined with Alpelisib in non-responding cells (Fig. 4C and D). However, the concomitant use of Alpelisib/SP600125, Alpelisib/Ralimetinib or Alpelisib/SCH772984 did not result in increased cytotoxicity or sensitivity to radio(chemo)therapy in non-responder compared to responder models (Fig. 4E).

Bioinformatics analysis nominated cell adhesion and cancer pathways as key determinants of combined PI3Kα/β1 integrin targeting in Alpelisib non-responders.

**Fig. 5.**
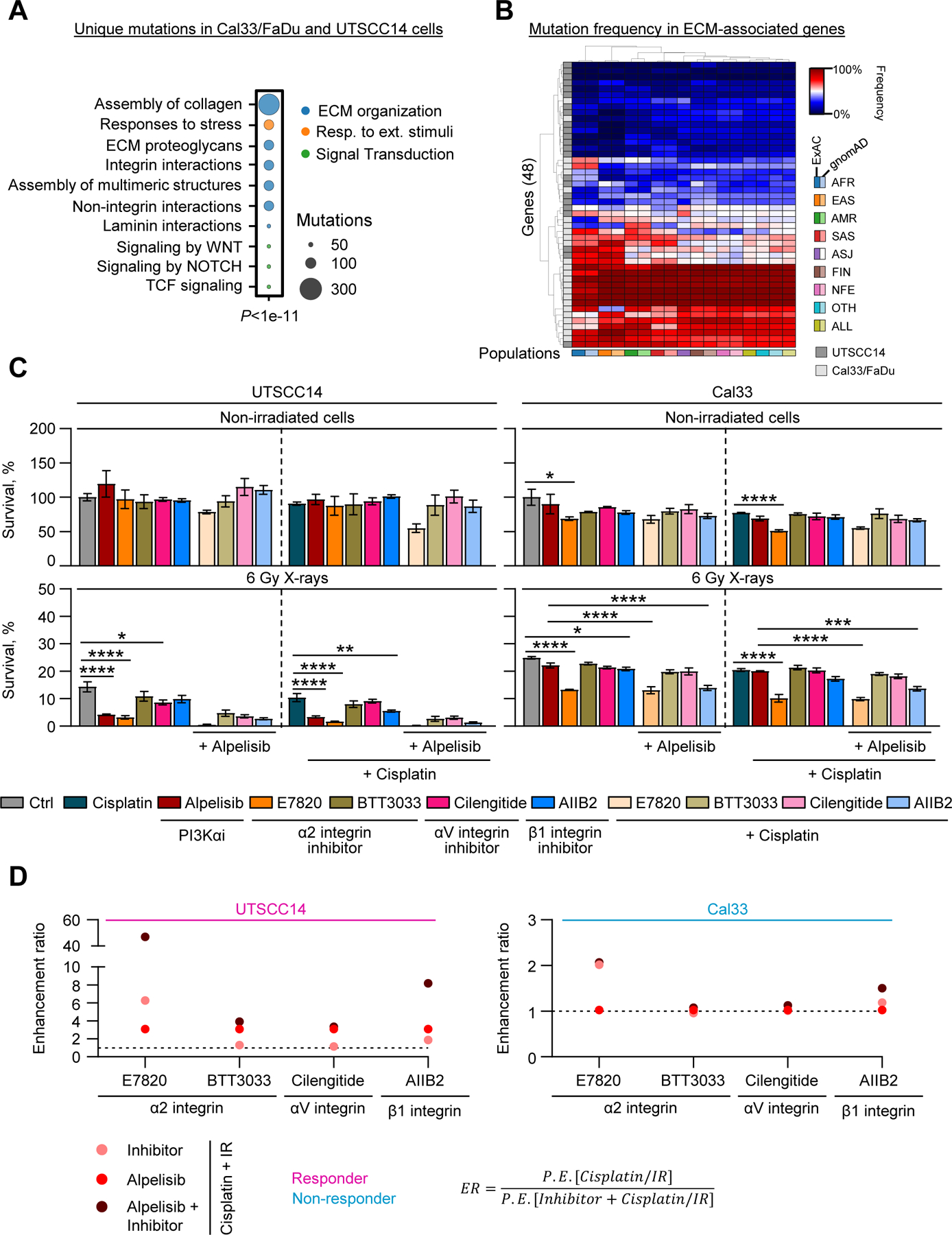
Co-targeting of PI3Kα and β1 integrin sensitizes HNSCC cells to radiochemotherapy. (A) Pathway enrichment analysis with unique mutated genes in responder (UTSCC14) and non-responder (Cal33, FaDu) cell models. (B) Heatmap illustrating frequency of unique mutations in ECM associated genes identified in HNSCC cells lines (UTSCC14, Cal33, FaDu across indicated human populations. AFR – Africans; EAS – East Asian; AMR – Central/South American; SAS – South Asian; ASJ – Ashkenazi Jewish; FIN – Finnish in Finland; NFE – non-Finnish European; OTH – other; ALL – all. (C) Basal clonogenic survival and clonogenic radiation survival of UTSCC14 and Cal33 cell lines treated with Alpelisib in combination with integrin inhibitors AIIB2 (β1 integrin), BTT3033, E7820 (α2 integrin), Cilengitide (αVβ3 and αVβ5 integrin) and/or Cisplatin/X-rays. Survival of untreated non-irradiated cells was set as 100%. Data represent mean ± SEM (*n* = 3). (D) Sensitizing effects in UTSCC14 and Cal33 cell lines upon combined treatments with Alpelisib and integrin inhibitors. Data are expressed as enhancement ratios (ER) calculated as indicated. Survival of cells treated with Cisplatin and X-rays was set as 1. Statistical analysis in C was performed using a one-way ANOVA with Sidak post hoc test; * , *P* < 0.05; * *, *P* < 0.01; * ** *, *P* < 0.0001; n.s. – non-significant.

Next, we conducted a more in-depth analysis of differential mutation profiles comparing responder (UTSCC14) and non-responder (Cal33, FaDu) models. We identified distinct mutations pertaining to cell-ECM interactions as a top enriched pathway for both responder and non-responder HNSCC models (Fig. 5A). Unique mutations in ECM-related genes were more frequently identified in non-responder models compared to responders, and these could be found in different human populations indicating a global and culture-independent presence of these mutations (Fig. 5B and Supplementary Table 1).

Given these findings, we next combined inhibition of PI3Kα with targeting ECM/adhesion receptors that have documented radio(chemo) sensitizing potential. Monotherapy using the α2 integrin (BTT3033, E7820), αV integrin (Cilengitide) and β1 integrin (AIIB2) inhibitors generally failed to mediate significant cytotoxicity in any tested treatment groups (Fig. 5C). In contrast, E7820 and AIIB2 monotherapy led to radiosensitization in Alpelisib responder (ER = 6.27 and = 1.88, respectively) and non-responder (ER = 2.02 and = 1.19, respectively) models (Fig. 5C, D). In combination with Alpelisib, the effects of E7820 and AIIB2 were further enhanced, with an ER of 47 for E7820 and 8.17 for AIIB2 in responders (Fig. 5C, D). In contrast, the radiochemosensitivity of non-responders was enhanced by AIIB2 with an ER of only 1.5 (Fig. 5C, D).

**Fig. 6.**
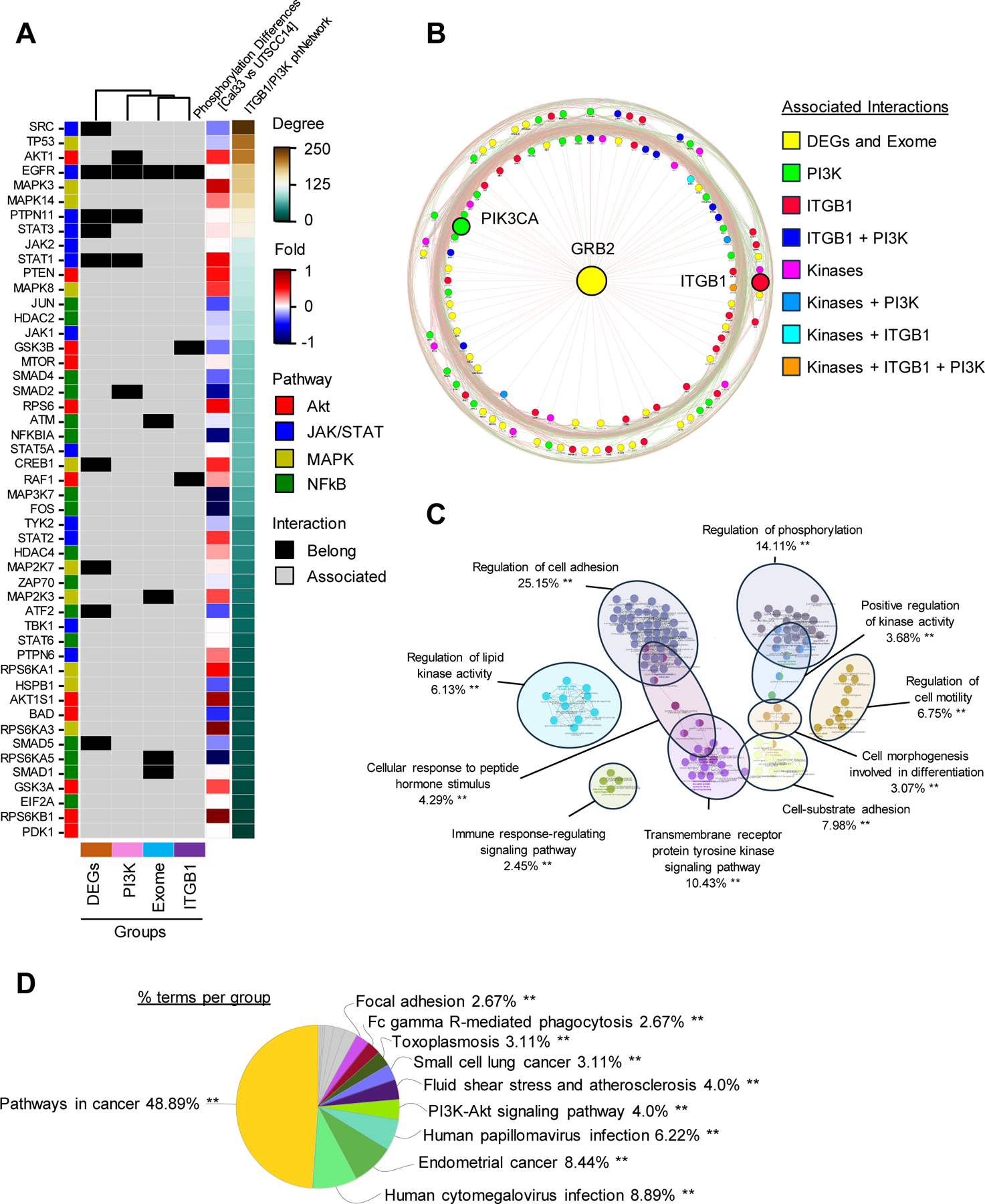
Adaptor protein Grb2 is predicted as an essential interactor bridging β1 integrin and PI3K signaling pathways in HNSCC cells. (A) Network analysis integrating WES data, DEGs, phospho-protein array and all interactors of β1 integrin and PI3Kα kinase. (B) Interaction network generated via GeneMANIA. The lines connect nodes with considered relationship. Radial structures indicate an increase of the interconnection enrichment from center to edge. Graphs illustrating functional enrichment analysis based on B generated using GO (C) and KEGG (D) databases.

Based on these observations showing a merely unchanged ER by Alpesilib for E7820 in contrast to AIIB2, we next aimed to bioinformatically identify critical kinases that might be responsible for overcoming the radiochemoresistance of Alpelisib in non-responders through PI3Kα/β1 integrin deactivation. We conducted an extensive network analysis integrating WES data, analyses of differentially expressed genes (DEG) for responding and non-responding HNSCC cells, our phospho-protein array data, and data on all known interactors of β1 integrin and PI3Kα (Fig. 6A). Through this comprehensive analysis, we discovered growth factor receptor-bound protein 2 (Grb2) as a central interactor bridging the pathways related to PI3Kα and β1 integrin (Fig. 6B). Further functional enrichment analysis highlighted genes within the final network as key determinants of cell adhesion and cancer pathways, shedding light on their potential regulatory roles in modulating cell behavior and tumorigenesis (Fig. 6C, D). Taken together, our data suggest a critical contribution of ECM proteins to the resistance of HNSCC cells to PI3Kα inhibition and conventional radio(chemo)therapy with β1 integrin/Grb2 as a putative key part of the adaptation mechanism.

## 4. Discussion

Intrinsic and acquired resistance of cancer cells can limit treatment efficacy to conventional as well as molecular targeted agents including PI3K-targeting therapies [4]. Therefore, identification of predictive biomarkers of response and resistance and understanding of underlying molecular mechanisms may ultimately lead to improved clinical outcomes for patients. In this study using eight 3D matrix-embedded HNSCC models, we show that (i) PI3K inhibition by Alpelisib, Copanlisib and AZD8186 but not Idelalisib promotes radio(chemo)sensitization in a heterogeneous fashion, (ii) Alpelisib is, among the three tested drugs, the most effective PI3K inhibitor and differently regulates MAPK signaling in responder and non-responder HNSCC cell models; (iii) unique mutations in ECM pathways are found in both Alpelisib responders and non-responders, but to a higher frequency in non-responders; (iv) double targeting of β1 integrin and PI3Kα kinase sensitizes non-responder HNSCC cells to radio(chemo)therapy, and (v) bioinformatic analyses reveals cell adhesion and cancer pathways to present key determinants of Alpelisib resistance in non-responders, whose impact can be diminished by combined PI3Kα/β1 integrin targeting.

Multiple studies demonstrated that the PI3K signaling axis is activated in various tumor entities including HNSCC and associated with increased tumor vascularization, metastasis and poor patient survival [3,4,7]. Therefore, targeting of PI3K in general or its isoforms represents a promising therapeutic direction. Indeed, in pre-clinical studies, PI3K inhibition significantly decreased proliferation, survival, cell cycle progression and migration of cancer cells [23–26]. In contrast, clinical testing of PI3K inhibitors applied as a monotherapy has shown limited efficacy to date. Moreover, pan-PI3K inhibitors were associated with a high risk of severe treatment-related toxicities [4,27]. Aiming for higher specificity, PI3K isoform-specific inhibitors have been developed based on PI3K mutations. The main alterations are mutations in *PIK3CA* and, less frequently, in *PIK3R1* (encoding the PI3K regulatory subunit p85α) [28]. Mutations in *PIK3CA*, predominantly the two most common *PIK3CA* somatic mutations, i.e. H1047R and E545K, have been proposed as predictive biomarkers for PI3K inhibitors including Alpelisib [6,29]. Importantly, analysis of the mutational profiles of our HNSCC cell model panel lacked correlation between *PIK3CA* mutations and Alpelisib-induced radio(chemo)sensitization. Furthermore, Alpelisib impaired the activity of the PI3K/Akt pathway to a similar degree in all cell models regardless of their Cisplatin/X-ray sensitivity. These findings are in line with recently published studies, which revealed that the *PIK3CA* mutation/amplification status fails to predict effectiveness of PI3Kα inhibitors against cancer cells [30–32]. In the context of acquired resistance, it has been shown that the c-Jun/Axl axis and an upregulation of insulin growth factor 2 (IGF2) are associated with a poor response to PI3Kα blockage in HNSCC and esophageal squamous cell carcinoma cells [33–35]. Additional studies highlighted an importance of cyclin D1 levels in resistance of cancer cells to PI3Kα inhibitors and revealed a synergistic anti-tumor effect upon combined targeting with CDK4/6 inhibitors [32,36,37]. In our study, we aimed to determine the responsiveness to Alpelisib in a larger HNSCC cell model panel and, by focusing on Alpelisib non-responding cell models, to uncover co-targeting strategies to overcome Alpelisib resistance and enhance radiochemosensitivity in these cell models.

It is well known that radio- and chemogenic DSB represent the most life-threatening DNA damage events [38]. Juvekar and co-authors revealed DNA damage induced by a depletion of the phosphorylated nucleoside pool in breast cancer cells treated with PI3K inhibitors [39]. In our study, Alpelisib impacted phosphorylation of DNA-PK and ATM but not ATR. However, these changes were neither significant relative to Cisplatin/X-rays nor between treated responder and non-responder HNSCC cell models. In line with these observations, no significant differences were detected in γH2AX, 53BP1 and Rad51 foci numbers indicating that Alpelisib enhances cellular radiosensitivity independent from DNA damage repair.

To continue our efforts to discover an effective targeting strategy to overcome Alpelisib resistance, we performed bioinformatics analyses and found that genes with unique mutations in responder (UTSCC14) and non-responder (Cal33, FaDu) cell models are associated with cell-ECM interactions. Based on our extensive studies on integrin targeting and its potential for radiochemosensitization of cancer cells, we tested various integrin inhibitors [10,40–44]. Without significant basal cytotoxicity of any of our integrin targeting approaches, we have only detected Alpelisib/Cisplatin/X-ray irradiation enhancement in non-responder HNSCC cells with concomitant inhibition of β1 integrin and PI3Kα.

Finally, by focusing on putative interactions between β1 integrin and PI3Kα, comprehensive bioinformatic analysis predicted an adaptor protein Grb2 as a competent of the molecular mechanisms underlying the synergistic response of HNSCC cells to β1 integrin/PI3Kα targeting. In accordance with our hypothesis, several previous studies indicated Grb2 as an essential component in the regulation of MAPK signaling, namely downregulation of Grb2 expression, reduced MAPK activation and impaired cancer cell survival [45,46]. Moreover, Grb2 is associated with β1 integrin [47] and overexpressed in various tumor entities [48].

In summary, our study shows a different cytotoxic and radiosensitizing potential of PI3K inhibitors. Furthermore, our data suggest that the cell adhesion receptor β1 integrin contributes to resistance to the PI3Kα inhibitor Alpelisib in a subset of HNSCC models via the adaptor protein Grb2. Therefore, we propose simultaneous targeting of β1 integrin and PI3Kα as a therapeutic option to improve the efficacy of PI3Kα inhibitors in combination with current standard therapy in HNSCC. Additional studies are needed to further elucidate these observations, particularly with regard to the identification of biomarkers for PI3K inhibitors and the development of agents targeting integrins.

## Supplementary Material

Suppl data for Fig 5

Suppl Figures

## Data Availability

Data will be made available on request.

## Abbreviations:

DEG: Differentially expressed genes
DSB: DNA double strand break
ECM: Extracellular matrix
ER: Enhancement ratio
GEO: Gene Expression Omnibus
HR: Homologous recombination
HNSCC: Head and neck squamous cell carcinoma
NHEJ: Non-homologous end joining
PI3K: Phosphoinositide 3-kinase
WES: Whole exome sequencing
