# Supplementary material for: β1 integrin mediates unresponsiveness to PI3Kα inhibition for radiochemosensitization of 3D HNSCC models
Source: Biomed Pharmacother. Author manuscript; Available in PMC 2024 Dec 9. (PMC11627550; doi:10.1016/j.biopha.2024.116217)
Supplement: Suppl Figures [file NIHMS2032510-supplement-Suppl_Figures.pdf]

Supplemental information

β1 integrin mediates unresponsiveness to PI3Kα inhibition for radiochemosensitization of 3D HNSCC models

Irina Korovina, Marc Elser, Olegs Borodins, Michael Seifert, Henning Willers, and Nils Cordes

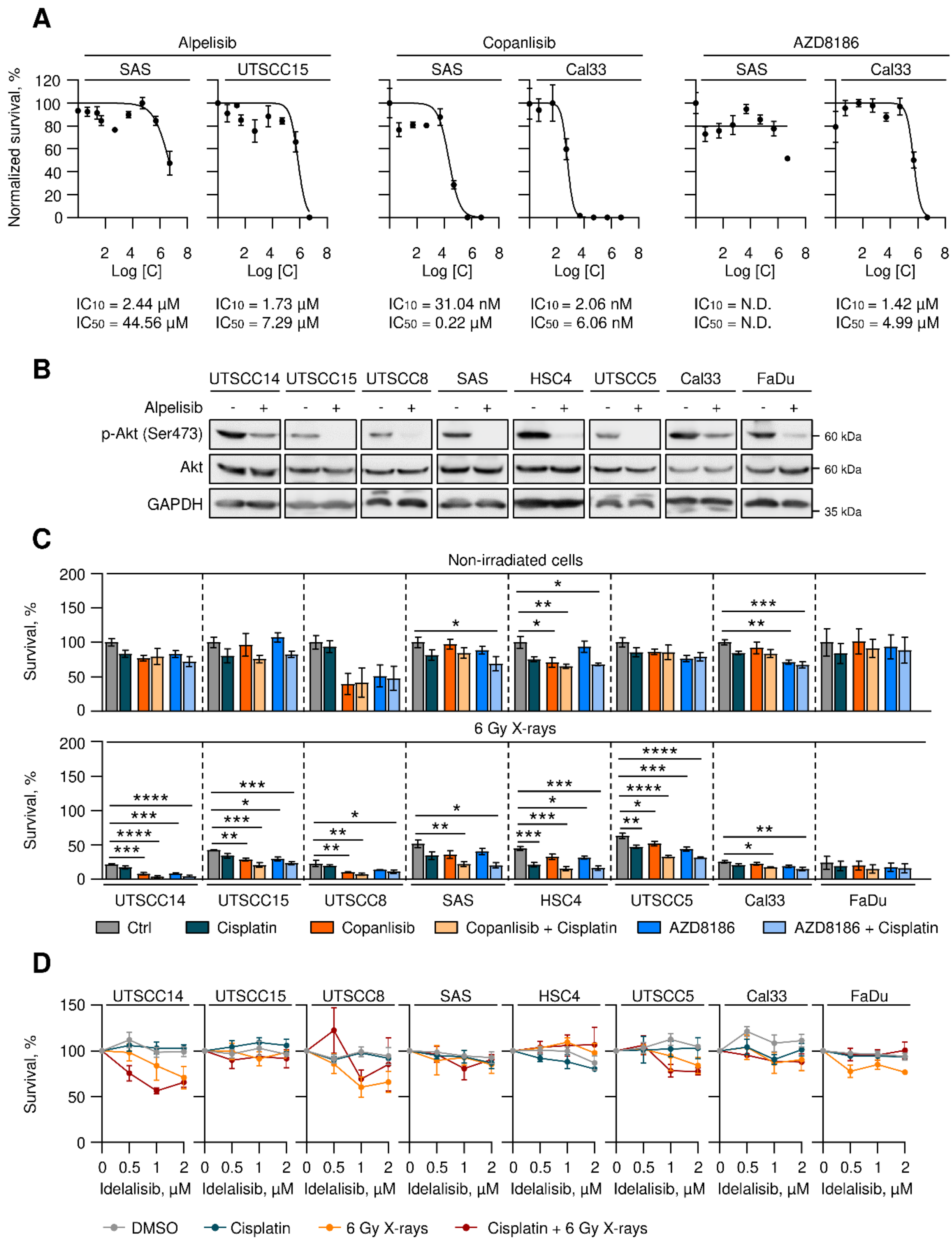

Fig. S1. Effects of PI3K inhibitors on survival of 3D lrECM HNSCC models. (A) Dose-response curves demonstrating 10% and 50% of maximal inhibitory concentration (IC10 and IC50) values of Alpelisib, Copanlisib and AZD8186. (B) Representative western blot images. GAPDH was used as a loading control. (C, D) Analysis of clonogenic survival in a panel of 3D

lrECM HNSCC models treated with PI3K inhibitors Copanlisib, AZD8186 or Idelalisib combined with Cisplatin. Survival of untreated non-irradiated cells was set as 100%. Data represent mean ± SEM of at least three independent experiments. Differences were compared using a one-way ANOVA with Dunnett post hoc test; *, $P<0.05$; **, $P<0.01$; ***, $P<0.001$.

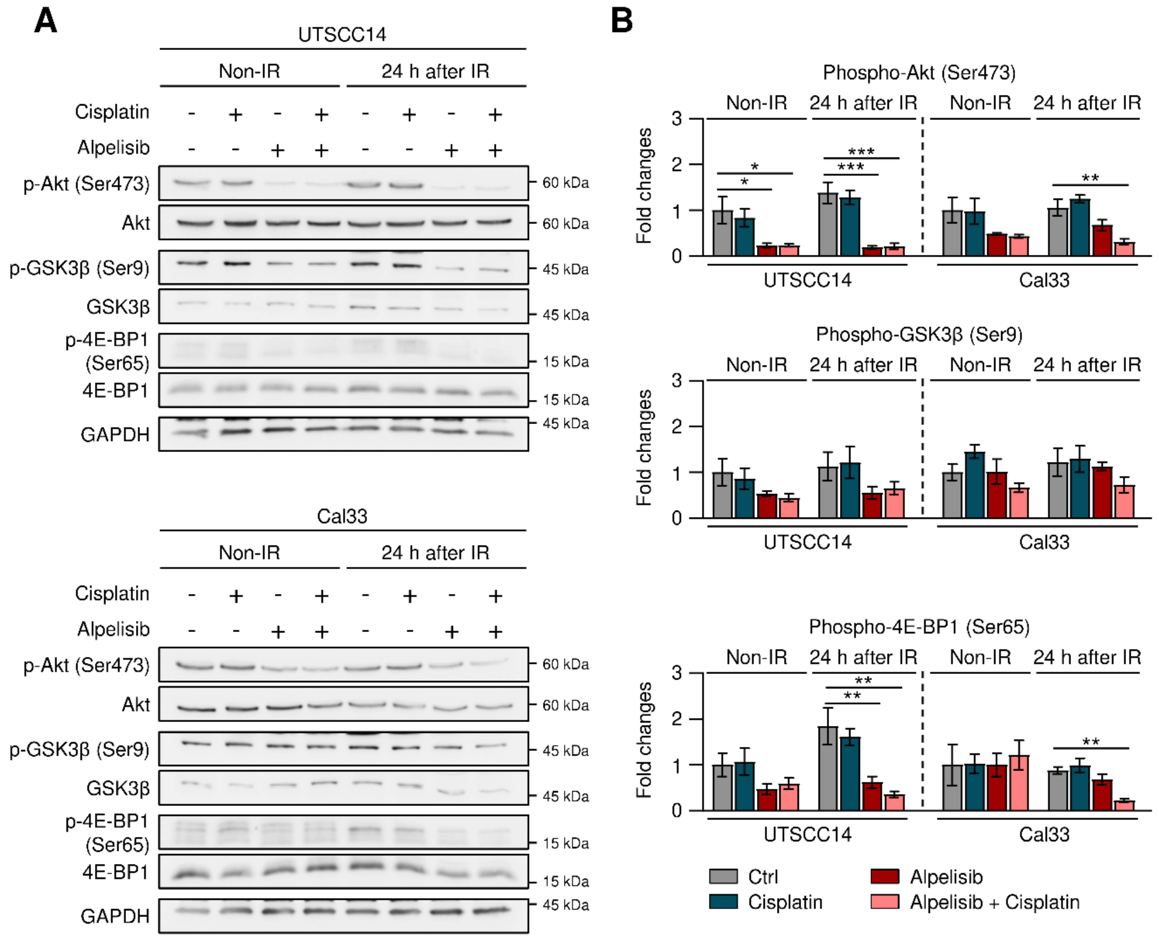

Fig. S2. Alpelisib reduces phosphorylation of Akt signaling pathway components in both responder and non-responder 3D lrECM HNSCC models. (A) Western blot analyses from whole cell lysates of UTSCC14 (responder) and Cal33 (non-responder) cell lines treated with Cisplatin, Alpelisib (DMSO as control) and 6 Gy X-rays (IR). (B) Densitometric quantifications of phosphorylated forms of indicated proteins. GAPDH was used as a loading control. Data presented as mean ± SEM (*n* = 4). Statistical analysis in B was performed using a one-way ANOVA with Dunnett post hoc test; *, *P*<0.05; **, *P*<0.01; ***, *P*<0.001.
